# Continuous glucose monitoring targets in type 1 diabetes pregnancy: every 5% time in range matters

**DOI:** 10.1007/s00125-019-4904-3

**Published:** 2019-06-03

**Authors:** Helen R. Murphy

**Affiliations:** Norwich Medical School, University of East Anglia, Floor 2, Bob Champion Research and Education Building, Norwich, NR4 7UQ UK

**Keywords:** Continuous glucose monitoring, Hyperglycaemia, Pregnancy, Type 1 diabetes

## Abstract

With randomised trial data confirming that continuous glucose monitoring (CGM) is associated with improvements in maternal glucose control and neonatal health outcomes, CGM is increasingly used in antenatal care. Across pregnancy, the ambition is to increase the CGM time in range (TIR), while reducing time above range (TAR), time below range (TBR) and glycaemic variability measures. Pregnant women with type 1 diabetes currently spend, on average, 50% (12 h), 55% (13 h) and 60% (14 h) in the target range of 3.5–7.8 mmol/l (63–140 mg/dl) during the first, second and third trimesters, respectively. Hyperglycaemia, as measured by TAR, reduces from 40% (10 h) to 33% (8 h) during the first to third trimester. A TIR of >70% (16 h, 48 min) and a TAR of <25% (6 h) is achieved only in the final weeks of pregnancy. CGM TBR data are particularly sensor dependent, but regardless of the threshold used for individual patients, spending ≥4% of time (1 h) below 3.5 mmol/l or ≥1% of time (15 min) below 3.0 mmol/l is not recommended. While maternal hyperglycaemia is a well-established risk factor for obstetric and neonatal complications, CGM-based risk factors are emerging. A 5% lower TIR and 5% higher TAR during the second and third trimesters is associated with increased risk of large for gestational age infants, neonatal hypoglycaemia and neonatal intensive care unit admissions. For optimal neonatal outcomes, women and clinicians should aim for a TIR of >70% (16 h, 48 min) and a TAR of <25% (6 h), from as early as possible during pregnancy.

In their observational cohort study of 186 pregnancies complicated by type 1 diabetes, Kristensen et al document fetal exposure to maternal glycaemia using detailed continuous glucose monitoring (CGM) measures [1]. Combining data from 92 real-time CGM (rt-CGM) and 94 intermittently viewed CGM (iCGM) ‘real-world’ users, they measured percentage of time spent in, above and below the target glucose range of 3.5–7.8 mmol/l (63–140 mg/dl), as well as mean glucose and glycaemic variability metrics throughout pregnancy. As expected, maternal glycaemia improved across gestation, with a decrease in mean glucose, HbA_1c_ and glycaemic variability. The authors confirm that the established clinical measures, HbA_1c_ and mean glucose, are good markers of the suboptimal glucose control associated with large for gestational age infants and neonatal complications. In addition, they describe the gestational changes in CGM measures. These data provide important new insights into the CGM measures associated with suboptimal maternal glucose control and risk of neonatal complications. They confirm the need for dynamic glycaemic metrics beyond HbA_1c_ and will inform the development of evidence-based CGM targets in type 1 diabetes pregnancy.

## CGM measures across gestation

Across pregnancy, the ambition is to increase the time in range (TIR) while reducing time above range (TAR), time below range (TBR) and glycaemic variability. With 84 women using CGM from before pregnancy, Kristensen et al demonstrate a substantial 15 percentage point increase in time in range (TIR 3.5–7.8 mmol/l) during the first trimester, rising from 40% TIR (10 h/day) in the early post-conception period to 55% TIR (13.2 h/day) by the end of the first trimester. There follows a striking lack of improvement across the second trimester, with a 5 percentage point increase bringing the third trimester TIR to 60% (14.4 h/day). This is mirrored by a reduction in first trimester time above target range (TAR >7.8 mmol/l), and minimal further reductions in the second and third trimesters.

Despite differences in patient populations, study design, statistical analyses, CGM systems and duration of sensor use, these Swedish CGM profiles are remarkably similar to those in the multicentre CONCEPTT trial [2]. The CONCEPTT control group (self-monitoring of blood glucose) had masked CGM profiles recorded at approximately 12, 24 and 34 weeks’ gestation, which are indistinguishable from the first, second and third trimester profiles described by Kristensen et al. Both report approximately 50% TIR 3.5–7.8 mmol/l and 40% TAR >7.8 mmol/l in the first trimester, improving to an average of 60% TIR 3.5–7.8 mmol/l and 33% TAR >7.8 mmol/l in the third trimester (Table 1). These data confirm the gap between our expectations of tight glucose control and the reality of achieving the CGM targets in standard antenatal care. The 2019 Advanced Technologies & Treatments for Diabetes (ATTD) consensus recommends targets for TIR of >70% (16 h, 48 min) and TAR of <25% (6 h), from as early as possible during pregnancy [3]. In the Swedish and CONCEPTT studies, women only achieved these targets towards the end of the third trimester, too late for optimal neonatal outcomes [1, 2].

**Table 1:** Patterns of glycaemia among pregnant CGM users with type 1 diabetes

Glucose measures	Kristensen et al, 2019 [1] iCGM or rt-CGM *N*=186^a^	Feig et al 2017 [2] rt-CGM *N*=108^a^	CGM target
Laboratory HbA_1c_, mmol/mol (%)			
Trimester 1	52 ± 10.5 (6.9 ± 1.0)	51 ± 7·3 (6·8 ± 0·7)	
Trimester 2	45 ± 7.9 (6.3 ± 0.7)	44 ± 6.5 (6.2 ± 0.5)	
Trimester 3	46 ± 7.6 (6.3 ± 0.7)	46 ± 6.7 (6·3 ± 0·6)	
CGM mean glucose, mmol/l			
Trimester 1	7.8 ± 1.4	7·3 ± 1·2	
Trimester 2	7.4 ± 1.2	7.6 ± 1.2	
Trimester 3	7.1 ± 1.1	6·7 ± 0·9	
TIR 3.5–7.8 mmol/l^b^ (%)			
Trimester 1	50 ± 14	52 ± 13	>70%
Trimester 2	55 ± 14	53 ± 15	>70%
Trimester 3	60 ± 13	68 ± 13	>70%
TAR >7.8 mmol/l^c^ (%)			
Trimester 1	43 ± 15	39 (28–49)	<25%
Trimester 2	38 ± 15	43 (29–54)	<25%
Trimester 3	34 ± 15	27 (19–37)	<25%
TBR <3.5 mmol/l^d^ (%)			
Trimester 1	7 ± 5	8 (4–14)	<4%
Trimester 2	7 ± 5	3 (1–6)	<4%
Trimester 3	6 ± 5	3 (1–6)	<4%
Glycaemic variability (% CV)^e^			
Trimester 1	40 ± 7	42 (38–47)	<36%
Trimester 2	38 ± 6	35 (31–39)	<36%
Trimester 3	36 ± 6	32 (28–37)	<36%

^a^Continuous glucose measures reported were obtained using rt-CGM and iCGM throughout pregnancy by Kristensen et al and at 10, 24 and 34 weeks in the group using rt-CGM by Feig et al

^b^TIR refers to % of time spent in range 3.5-7.8 mmol/l (63-140 mg/dl). The TIR target 3.5-7.8 mmol/l (63-140 mg/dl) proposed by the ATTD consensus group is >70% (16 h 48 min) in type 1 diabetes pregnancy

^c^TAR refers to % of time spent >7.8 mmol/l (>140 mg/dl). The TAR target >7.8 mmol/l (>140 mg/dl) proposed by the ATTD consensus group is <25% (6 h) in type 1 diabetes pregnancy. Values are means ± SD or median (interquartile range) as reported in the publications by Kristensen et al [1] and Feig et al [2]

^d^TBR refers to the % of time spent <3.5 mmol/l (<70 mg/dl). The TBR target <3.5 mmol/l (<70 mg/dl) proposed by the ATTD consensus group is <4% (1 h) in type 1 diabetes pregnancy. rt-CGM users spent less time below 3.5 mmol/l compared with iCGM users in the study by Kristensen et al [1]

^e^Glucose coefficient of variation (CV) thresholds for stable ≤36% and unstable >36% are based on thresholds outside of pregnancy

## CGM measures in relation to HbA_1c_

The limitations of HbA_1c_ when evaluating individual glucose control are well recognised [4]. Among women without diabetes, HbA_1c_ is lower during pregnancy, as a result of lower mean glucose [5] and artefactual lowering that is unrelated to maternal glycaemia [6]. During early pregnancy, artefactual lowering is attributed to increased erythropoiesis and shortened red cell life span [5, 6]. We previously described gestational reductions of up to 11 mol/mol (1%) HbA_1c_, without improvement in self-monitored glucose levels [7]. Women with higher HbA_1c_ values have the largest HbA_1c_ reductions, which can be falsely reassuring in those with very poor control [7].

Astute readers of the paper by Kristensen et al will notice that maternal HbA_1c_ levels remained unchanged from the second to the third trimester despite a 0.3 mmol/l reduction in mean glucose and a 5% percentage point increase in TIR (from 55% to 60%). Similar discrepancies between third trimester HbA_1c_ and improving CGM glycaemic measures (TIR, TAR) were observed in CONCEPTT. Twice as many CONCEPTT participants achieved target HbA_1c_ compared with target TIR, suggesting that a TIR of >70% is a more ambitious goal.

A formula for converting CGM-derived glucose mean glucose into an estimated HbA_1c_ during pregnancy has been proposed [8]. To avoid confusion with laboratory HbA_1c_ assays, the CGM estimated HbA_1c_ is now more appropriately referred to as a glucose management indicator [9]. Law et al analysed data from two early randomised trials of intermittent retrospective or rt-CGM [10, 11], and these results suggested that, to minimise fetal overgrowth, clinicians should focus on mean CGM glucose levels, with a recommended target of 6.4–6.7 mmol/l (115–120 mg/dl) [12]. Notably, this target was not achieved, either in the early [10, 11] or in the recent CGM studies [1, 2] until the final weeks of pregnancy.

## CGM measures in relation to fetal and neonatal outcomes

During organogenesis, the developing fetus is particularly susceptible to maternal glucose excursions, and peri-conception hyperglycaemia as measured by HbA_1c_ is strongly associated with increased risk for major congenital anomaly, stillbirth and neonatal death [13–16]. Large population-based CGM studies are required to confirm the associations between CGM measures and serious adverse pregnancy outcomes. However, for commonly occurring obstetric and neonatal complications, including large for gestational age (LGA) infants, neonatal intensive care unit (NICU) admissions and neonatal hypoglycaemia [13–16], CGM-based risk markers are emerging.

Kristensen et al found that a 5–7% lower TIR during the second and third trimesters was associated with increased risk of LGA and neonatal outcomes, including macrosomia, shoulder dystocia, neonatal hypoglycaemia or NICU admissions of >24 h duration. Mothers of infants with vs without LGA had lower TIR during the second (52% vs 58%) and third trimesters (58% vs 62%). Likewise, mothers of infants with complications of macrosomia, shoulder dystocia, neonatal hypoglycaemia or NICU admissions had a 5–6% lower TIR (TIR 52% vs 57% and TIR 56% vs 62%) during the second and third trimesters.

In CONCEPTT, we also found that rt-CGM users (compared with self-monitoring) achieved a 5–7% higher TIR in the second and third trimesters, and this was associated with a halving in the odds ratio for LGA, neonatal hypoglycaemia and NICU admissions of >24 h duration [2]. Taken together, these data indicate that relatively small (5%) increments in TIR are associated with clinically relevant improvements in neonatal health outcomes. Importantly, TIR increments are attainable and not influenced by gestational changes in erythropoiesis, red cell life span or iron deficiency.

## CGM measures in relation to maternal hypoglycaemia

Outside of pregnancy, percentage of TBR <3.9 mmol/l and TBR <3.0 mmol/l have both been associated with clinical episodes of severe hypoglycaemia [17]. During normal healthy pregnancy, glucose levels are approximately 20% lower [18]. A TBR threshold of less than 3.5 mmol/l, was used in the Swedish and CONCEPTT studies [1, 2]. Kristensen et al demonstrate a substantial (almost twofold) increase in the percentage of time spent below 3.5 mmol/l, starting from 6 weeks and peaking at 12–16 weeks’ gestation. This is consistent with the well-recognised time-frame associated with maternal risk of severe hypoglycaemia [19]. As maternal hypoglycaemia is the rate-limiting factor for achieving tight glycaemic targets in early pregnancy, these data suggest that the target of minimising time spent below 3.5 mmol/l to less than 4% (1 h per day) is most challenging until around 16 weeks’ gestation.

Kristensen et al did not report severe hypoglycaemia events. In CONCEPTT there were too few severe hypoglycaemia episodes, (14 with pump, 13 with multiple daily injection) to examine associations with CGM time below range thresholds. The time spent below 3.5 mmol/l halved both in insulin pump and multiple daily injection users (from 6% to 3% and from 8% to 4%) from 12 to 34 weeks’ gestation [20]. Taken together, the Swedish and CONCEPTT data suggest that, with contemporary antenatal care, the proposed Advanced Technologies & Treatments for Diabetes (ATTD) consensus recommendation of not more than 4% of time (1 h/day) spent below 3.5 mmol/l is safely achievable, especially after 16 weeks. As CGM time below range data are highly skewed, increasing the lower threshold to 3.9 mmol/l, is associated with a substantial two- to threefold increase in time spent below range. We previously reported up to 15% of time (3 h 45 min) below 3.9 mmol/l across type 1 diabetes pregnancy. Kristensen et al report approximately 10% of time (2.4 h/day) spent below 3.5 mmol/l in iCGM users. CGM data on time below range are particularly sensor dependent, but regardless of the threshold used for individual patients in clinical practice, spending ≥4% of time (1 h/day) below 3.5 mmol/l or ≥1% of time (15 min/day) below 3.0 mmol/l is not recommended.

Once maternal insulin sensitivity starts to decline, typically around 18–20 weeks’ gestation, the challenge is to minimise postprandial hyperglycaemia [21]. While modern insulin analogues [22, 23], rt-CGM [24] and hybrid closed-loop systems [25] all contribute to reducing hypoglycaemia in pregnancy, optimising hyperglycaemia requires early prandial bolus insulin (30–45 min before meals in late gestation) [26] and meticulous attention to dietary intake for meals and snacks [27].

## iCGM or rt-CGM?

The factory-calibrated Freestyle Libre iCGM used in the study by Kerstensen et al [1] is more affordable and free from the alarms that frustrate rt-CGM users [28]. A study among 74 pregnant women (39 GDM, 24 type 1, 11 type 2 diabetes) led to a Conformité Européenne (CE) mark meaning that it complies with health, safety and environmental protection standards. It is the first sensor with a CE mark specifically for use during pregnancy. The accuracy of the Freestyle Libre sensor is acceptable and comparable between pregnant and non-pregnant users [29]. We found similar agreement between rt-CGM sensor accuracy in early and late gestation and between pregnant and non-pregnant users [30]. Thus, it is generally accepted that the sensor accuracy of iCGM and/or rt-CGM is not affected by gestational physiology.

In the study by Kristensen et al, women with iCGM appeared to spend more time below range at all gestational ages. Whether this relates to the diabetes self-management behaviours of rt-CGM users to prevent and/or avoid hypoglycaemia or to sensor accuracy of iCGM at lower glucose concentrations is unknown. All CGM sensors are less reliable at glucose levels below 3.5 mmol/l. However, iCGM users spent strikingly large proportion of time (10%) below target during the latter part of the first and third trimesters (Fig. 1c in [1]). The apparently high rates of time below target reported during the third trimester are unexpected and if women are then snacking to avoid perceived hypoglycaemia this could potentially undermine efforts to optimise glucose control in late pregnancy.

Despite baseline differences in maternal characteristics (longer duration of type 1 diabetes, a higher proportion of insulin pump users among the women using rt-CGM), Kristensen et al found that rates of LGA and neonatal composite outcome were comparable between iCGM and rt-CGM users. An adequately powered, large scale, randomised controlled trial would be required to demonstrate that iCGM is non-inferior to rt-CGM. Meanwhile, the randomised trial evidence supports rt-CGM use for improving neonatal health outcomes [2].

## Clinical CGM targets in pregnant individuals with type 1 diabetes

The patterns of glycaemia during pregnancy have been studied for over five decades using laboratory and capillary glucose measures. These informed simple fasting, pre-meal and post-meal glucose targets in pregnant women with and without diabetes [31]. The patterns of glycaemia using CGM are more complex and require evidence-based CGM metrics based on pregnancy outcomes. As noted by Kristensen et al, day-to-day glucose control was suboptimal despite use of iCGM or rt-CGM. We still have much to learn on how best to adjust the timing and dosing of insulin, physical activity and dietary intake in relation to CGM data. The incorporation of clinical CGM based-targets may help users and clinicians to interpret CGM data and to agree specific, measurable and potentially attainable CGM targets over a typical 2–4 week antenatal clinic time-frame. Goal setting based on CGM-based targets could help overcome the current lack of glycaemic improvement in the second and third trimesters.

CGM measures give insights into direct fetal exposure to maternal glycaemia and, unlike HbA_1c_, are not subject to changes in gestational physiology. To optimise day-to-day glucose control, women and clinicians should focus their attention on the dynamic CGM measures of fetal glucose exposure. The ATTD consensus CGM target recommendations suggest aiming for a TIR for 3.5–7.8 mmol/l of >70% (16 h, 48 min), TAR for >7.8 mmol/l of <25% (6 h) and TBR for <3.5 mmol/l of <4% (1 h) and TBR for <3.0 mmol/l of <1% (15 min) [3].

This ambitious target of >70% TIR is currently only reached in the final 3–4 weeks of type 1 diabetes pregnancy, which is too late for optimal neonatal outcomes. In CONCEPTT, only 10% of women achieved a TIR of >70% during the first and second trimesters, rising to 30% at 34 weeks. Almost twice as many achieved target HbA_1c_ levels. For optimal obstetric and neonatal outcomes, women and clinicians should aim to reach a TIR of >70% (16 h, 48 min) and a TAR of <25% (6 h), from as early as possible during pregnancy. Women who cannot achieve the TIR target of >70% in the second and early third trimester should be encouraged that a 5% increase in TIR is associated with clinically relevant improvements in neonatal health.

## Abbreviations

ATTD: Advanced Technologies & Treatments for Diabetes
CE: Conformité Européenne
CGM: Continuous glucose monitoring
iCGM: Intermittently viewed CGM
LGA: Large for gestational age
NICU: Neonatal intensive care unit
rt-CGM: Real-time CGM
TAR: Time above range
TBR: Time below range
TIR: Time in range
